# Retinoic acid treated human dendritic cells induce T regulatory cells via the expression of CD141 and GARP which is impaired with age

**DOI:** 10.18632/aging.100973

**Published:** 2016-05-30

**Authors:** Sudhanshu Agrawal, Sreerupa Ganguly, Alexander Tran, Padmaja Sundaram, Anshu Agrawal

**Affiliations:** ^1^ Division of Basic and Clinical Immunology, Department of Medicine, University of California, Irvine, CA 92697, USA

**Keywords:** dendritic cells, Retinoic Acid, GARP, aging, T regulatory cells

## Abstract

Aged subjects display increased susceptibility to mucosal diseases. Retinoic Acid (RA) plays a major role in inducing tolerance in the mucosa. RA acts on Dendritic cells (DCs) to induce mucosal tolerance. Here we compared the response of DCs from aged and young individuals to RA with a view to understand the role of DCs in age-associated increased susceptibility to mucosal diseases. Our investigations revealed that compared to young DCs, RA stimulated DCs from aged subjects are defective in inducing IL-10 and T regulatory cells. Examinations of the underlying mechanisms indicated that RA exposure led to the upregulation of CD141 and GARP on DCs which rendered the DCs tolerogenic. CD141^hi^, GARP^+^ DCs displayed enhanced capacity to induce T regulatory cells compared to CD141^lo^ and GARP^−^ DCs. Unlike RA stimulated DCs from young, DCs from aged subjects exhibited diminished upregulation of both CD141 and GARP. The percentage of DCs expressing CD141 and GARP on RA treatment was significantly reduced in DCs from aged individuals. Furthermore, the remaining CD141^hi^, GARP^+^ DCs from aged individuals were also deficient in inducing T regs. In summary, reduced response of aged DCs to RA enhances mucosal inflammation in the elderly, increasing their susceptibility to mucosal diseases.

## INTRODUCTION

Elderly are highly susceptible to mucosal infections and diseases. Respiratory mucosal infections such as influenza and pneumonia are often more severe and prolonged in the aged population[1, 2]. Asthma and COPD are also more prevalent in the elderly[3, 4]. Similar increases in incidences and severity of infections of the gastro-intestinal tract are also observed. For example, aged subjects over 65 years display significantly higher morbidity and mortality to clostridium difficile infection, the most frequent cause of nosocomial diarrhea[5]. Advancing age is also a major risk factor for colon cancer[6]. In spite of the clinical observation that old age is one of the major risk factors for these diseases, the alterations of the mucosal innate responses that predispose the elderly to infections are not well understood. There is a paucity of information regarding the contribution of the dendritic cells (DCs) which are initiators and regulators of the immune response[7].

Mucosal surfaces are exposed continuously to a broad range of antigens during respiration and food intake. Most of these antigens are harmless, non-pathogenic antigens. Immune cells of the mucosa have to be equipped to deal with and distinguish between innocuous and pathogenic antigens. Emerging evidence indicates that DCs play a pivotal role in mediating mucosal tolerance against harmless antigens while mounting inflammatory responses against harmful pathogens [8]. DCs are present below the epithelial cell layer and survey the airway and intestinal lumen via extension of dendrites [9]. Antigens are taken up the DCs in the mucosal environment but the activation of DCs is prevented by immunosuppressive factors in the mucosa. Vitamin A metabolite, Retinoic Acid (RA) is found in abundance in the mucosa and plays a crucial role in maintaining tolerance at these surfaces [10, 11]. RA is a lipophilic molecule that controls the activity of a constellation of genes via binding to nuclear receptors [12]. It has been demonstrated to act on DCs to render them tolerogenic and induce Foxp3^+^ regulatory T cells [11, 13]. RA therefore influences DCs to adopt an inhibitory phenotype to prevent response against harmless antigens. This is extremely important in preventing inflammation and maintaining lung and gut homeostasis as increased sterile inflammation in the mucosa is major risk factor for infections and cancers [2, 14, 15].

Besides generating tolerance, RA produced by DCs is also essential for induction of immunity to pathogens in the mucosa. RA induces gut homing effector T cells and IgA producing B cells [16, 17]. Strikingly, there is a scarcity of information regarding the effect of RA on DCs in the elderly. Our previous studies indicate that the functions of DCs are altered substantially with age [18-22]. DCs from aged subjects tend to display enhanced inflammatory responses and reduced capacity to regulate inflammation. Therefore, we investigated the response of DCs from aged to RA with a view to understand the role of DCs in age-associated increased susceptibility to mucosal diseases.

Here we identified novel mechanisms of Treg induction by RA stimulated DCs which are compromised in the elderly. We demonstrate that CD141 and GARP expression on DCs induced by RA stimulation played an essential role in inducing Tregs. Furthermore, both the expression of these molecules and capacity to induce Tregs was impaired in RA treated DCs from the elderly. This is the first report which demonstrates that altered function of DCs from elderly impairs mucosal tolerance and thus enhances the inflammation in the mucosa. These findings provide an important basis for the development of novel therapeutic strategies to treat mucosal diseases in the elderly.

## RESULTS

### RA treated DCs from aged subjects are impaired in their ability to induce T regulatory cells

**Figure 1:**
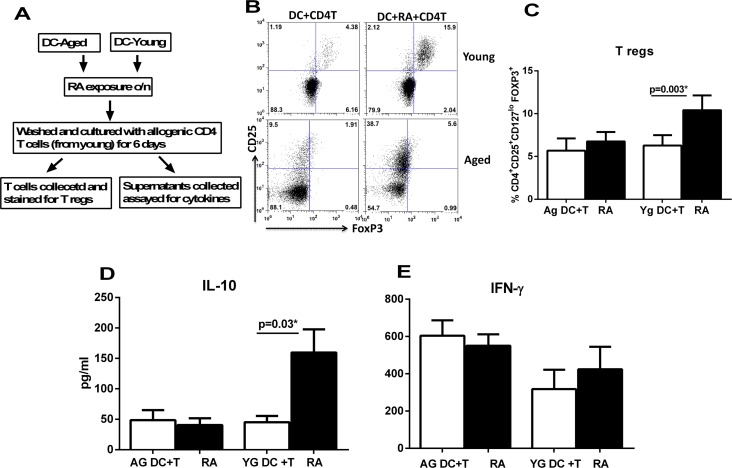
DCs from elderly are compromised in their ability to induce T regulatory cells after RA treatment DCs from aged and young subjects were stimulated with RA and subsequently cultured with CD4 T cells. T cell priming was assayed. (**A**) Flow chart for experimental set up. (**B**) Dot plot depicts the percentage of CD4^+^CD25^+^CD127^lo^ FoxP3^+^ T reg cells after 5 days co-culture of DCs with CD4 T cells. Figures are representative of 10 such experiments with different aged and young subjects. (**C)** Bar graphs Mean +/− S.E. of the percentage of CD4^+^CD25^+^CD127^lo^ FoxP3^+^ T reg cells. Bar graphs depict the levels of cytokines secreted by T cells in the DC-T cell co-culture. (**D**) IL-10; **E.** IFN-γ. Figures are mean ^+^/− S.E. of 10 experiments with different aged and young subjects.

Reports in the literature suggest that RA secreted by epithelial cells acts on DCs to render them tolerogenic and programs them to induce T regs [10, 13]. Our previous studies have suggested that DCs from aged subjects display enhanced basal level of inflammation and reduced capacity to maintain tolerance to self-antigens [20]. Here we examined whether the aged DCs are also impaired in their response to mucosal tolerogenic signals such as RA. Initial studies were performed to determine the optimal concentration of RA which can affect the function of DCs without causing cell death. Concentrations of 0.1-10μM/ml of RA were used and 1μM/ml was found to be optimal (Supplementary fig.1). Higher concentrations were toxic to DCs. Immature DCs from aged and young subjects were exposed to RA at a concentration of 1μM/ml. After overnight exposure, the DCs were collected and cultured with allogenic CD4 T cells from young individuals (to rule out any T cell defect) and induction of T regs was determined by staining for transcription factor, FoxP3 in CD4^+^CD25^+^CD127^lo^ gated cells. Flow chart for the experimental set up is provided in Figure 1A. Culture of aged and young DCs exposed to RA resulted in significant induction (p<0.05) of Tregs compared to unexposed DCs. Nevertheless, the capacity of RA exposed DCs from aged subjects to induce Tregs was significantly impacted (p<0.05) (Figure 1B & C) compared to their young counterparts. These results were further confirmed by cytokine assays where also, the T cells cultured with DCs from aged subjects displayed significantly reduced (p<0.05) IL-10 secretion (Figure 1D). The secretion of IFN-γ by T cells was not significantly (p>0.05) affected by stimulation of DCs with RA (Figure 1E). However, CD4^+^T cells cultured with unstimulated DCs from aged secreted significantly (p<0.05) higher levels of IFN-γ compared to young indicating a higher basal level of activation. This is in keeping with our previous studies [21, 23]. These results demonstrate that DCs from aged subjects display reduced capacity to induce T regs in response to RA and are therefore defective in their response to RA compared to young subjects.

### RA exposure upregulated the expression CD141 and LRRC32/GARP on DCs which is defective in DCs from aged subjects

Next, we determined the mechanisms responsible for reduced induction of Tregs by RA exposed DCs from aged subjects. Recent murine studies suggest that the CD103**^+^** DC subset (CD141 in humans) in the mucosa is responsible for inducing T regs [13, 24]. Murine studies also suggest that CD103 expression is upregulated on DCs in the mucosa via the action of RA on DCs [13]. Therefore, we investigated whether exposure of DCs to RA led to the induction of CD103. In addition to CD103, the upregulation of CD141, Glycoprotein A Repetitions Predominant (GARP)/Leucine Rich Repeat Containing 32 (LRRC32) and Latency associated peptide (LAP/TGF-β1) was also determined. CD141 was chosen because CD141^+^ DCs are reported to be the human homolog of CD103^+^ DCs [25]. GARP was chosen because it was found to be significantly downregulated in DCs from aged compared to DCs from young subjects in our gene array data [26]. GARP expression has previously been reported on platelets and T regulatory cells where it plays an important role in tethering latent TGF-β on the surface [27, 28]. More recently GARP expression has also been observed on mesenchymal stem cells (MSCs) [29] where it is involved in inducing tolerance in T cells. Little is known about whether GARP is expressed on other cells including DCs, and if it is, whether GARP plays any role in regulating the function of these cells. Studies suggest that GARP is usually expressed together with Latency associated peptide (LAP) on the surface of activated FoxP3+ Tregs and platelets [27, 28]. Therefore, we also determined the expression of LAP on RA treated DCs.

**Figure 2:**
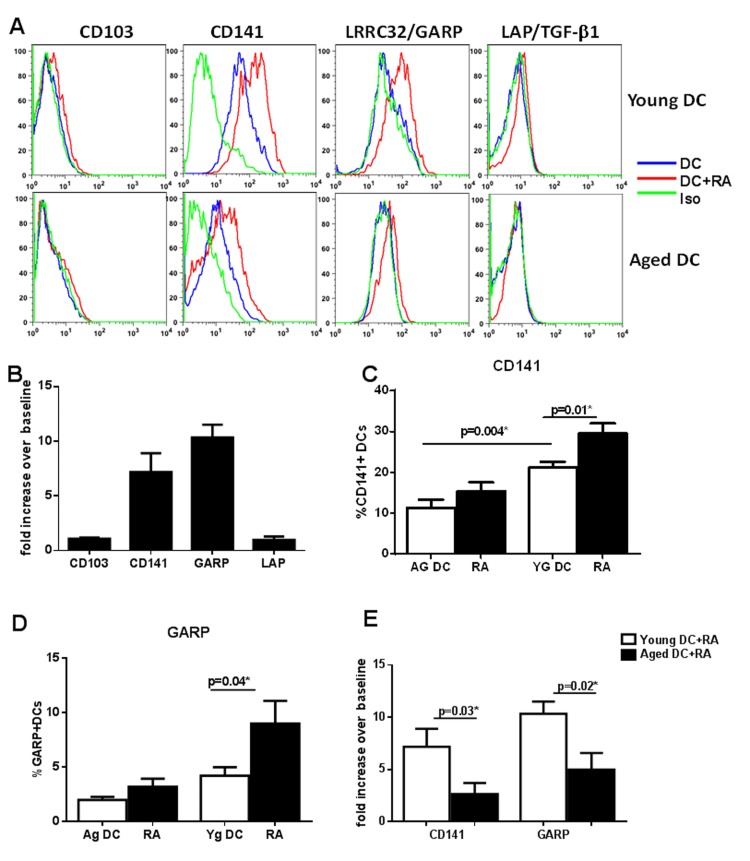
RA exposure upregulated the expression CD141 and LRRC32/GARP on DCs which is defective in DCs from aged subjects DCs from aged and young subjects were stimulated with RA at 1 μM and upregulation of the expression of CD103, CD141, GARP/LRRC32 and LAP/TGF-β1 was determined. (**A**) Histograms depict the expression of these molecules on aged and young DCs as determined by flow cytometry. (**B)** Bar graph depicts the upregulation of genes for above mentioned molecules on DCs 2h post stimulation with RA. Bar graph depicts the percentage of – (**C**) CD141^+^ (**D**) GARP^+^ DCs in aged and young subjects before and after stimulation with RA. (**E**) Bar graph depicts the upregulation of genes for CD141 and GARP on aged and young DCs 2h post stimulation with RA. Figures are mean ^+^/− S.E. of 15 experiments with different young subjects.

The culture of DCs in the presence of RA resulted in significant (p<0.05) upregulation in the expression of CD141 and GARP (Figure 2A). Low level increase in LAP and CD103 expression was also observed which was not significant (p>0.05). Gene expression results via PCR concurred with the surface expression results (Figure 2B). Remarkably, the expression of both CD141 and GARP was significantly reduced (p<0.05) both at the protein and gene level in DCs from aged subjects verses the young (Figure 2A, C-E). There was no major change in the expression of CD103 and LAP after co-culture and the expression was comparable between the two age groups (Figure 2A).

Together these data demonstrate that the upregulation of CD141 and GARP as well as induction of T regs in response to RA is impaired in DCs from aged subjects.

### CD141 and GARP Co-localize in RA treated DCs

**Figure 3:**
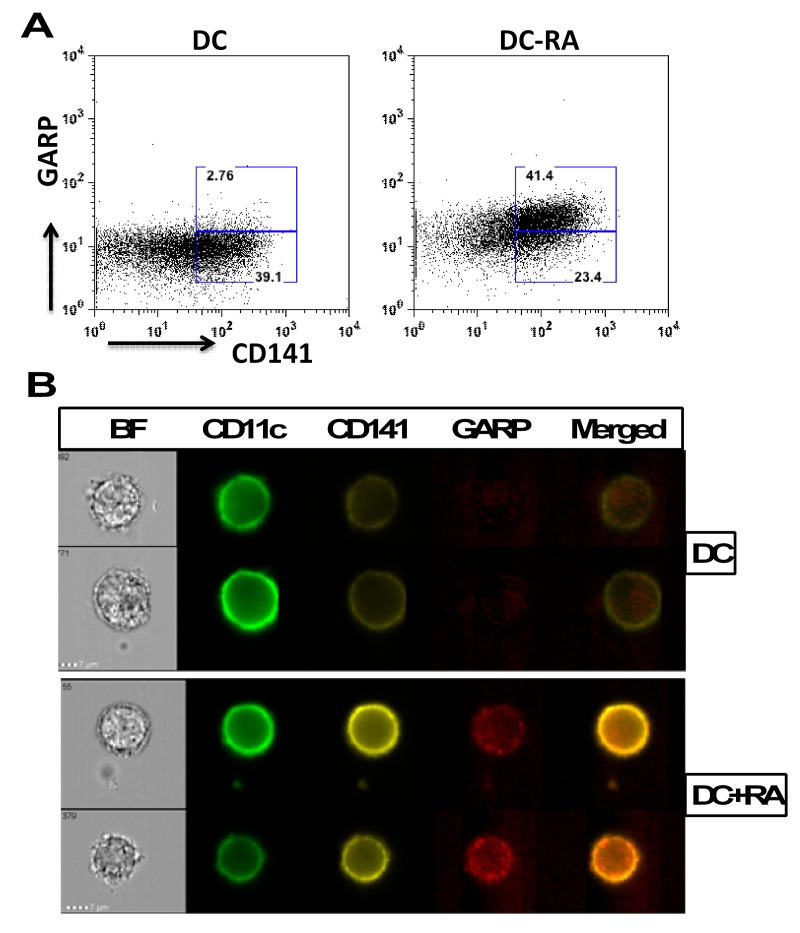
CD141 and GARP co-localize in RA treated DCs DCs from aged and young subjects were treated overnight with RA. The expression of GARP and CD141 on gated CD11c DCs was determined by flow cytometry. (**A**) Dot plot depicts the expression of CD141 and GARP on DCs and the percentages of CD141^hi^GARP^+^ and CD141^lo^ GARP^−^ DCs. (**B**) Images depict the co-localization of CD141 and GARP after RA exposure of DCs as determined by Imagestream. BF-brightfield image; Merged is CD141/GARP. Representative of 3 such experiments.

Since RA treatment of DCs led to the exposure of both CD141 and GARP, we determined whether the GARP and CD141 are co-expressed on the same cells. As is evident from figure 3A, GARP is expressed mainly on CD141 high cells. Further confirmation for colocalization of CD141 and GARP in DCs was obtained by Imagestream flow cytometer which provides microscopic images as well as flow data. Similar to flow data, image data also suggested that CD141 and GARP expression is upregulated on DCs by RA and the two molecules co-localize (Figure 3B).

### Expression of CD141 and GARP on DCs renders them tolerogenic enhancing their capacity to induce T regulatory cells

**Figure 4:**
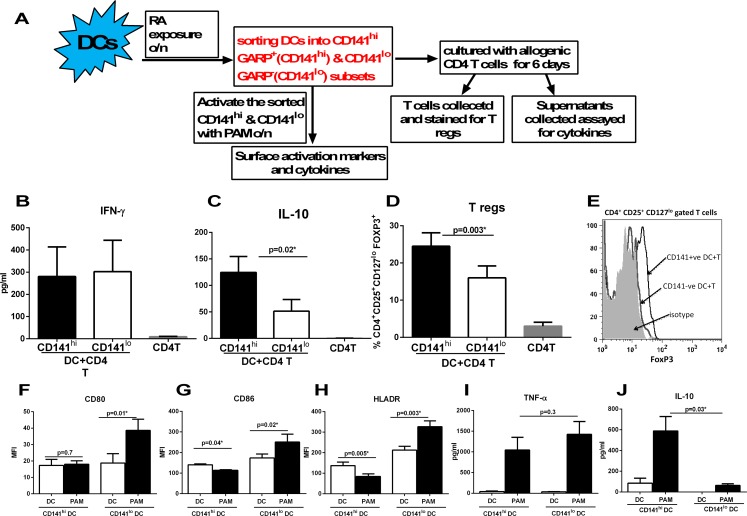
Expression of CD141 and GARP on DCs renders them tolerogenic enhancing their capacity to induce T regulatory cells DCs from young subjects were stimulated with RA at 1 μM for 24h. RA- treated DCs were sorted into CD141^hi^, GARP^+^ (CD141^hi^) and CD141^lo^, GARP^−^ (CD141^lo^) populations and cultured with allogenic CD4 T cells. (**A**) Flow chart for experimental set up. Bar graphs depict- (**B**) the level of IFN-γsecretion; (**C**) IL-10 secretion; (**D**) the percentage of CD4^+^CD25^+^CD127^lo^ FoxP3^+^ T reg cells; (**E**) Histogram depicts the expression of FoxP3 on CD4^+^CD25^+^ CD127^lo^ gated cells. Figures are mean ^+^/− S.E. of 5 different experiments. Purified CD141^hi^ and CD141^lo^ populations of DCs were stimulated with PAM for 24h. The expression of expression markers and cytokine secretion was determined. Bar graphs depict the Mean Fluorescence Intensity (MFI) of- (**F**) CD80; (**G**) CD86; (**H**) HLADR. Bar graphs depict the levels of (**I**) TNF-α; (**J**) IL-10. Figures are mean ^+^/− S.E. of 4 different experiments.

Exposure to RA led to the upregulation of CD141 and GARP on DCs. These DCs also induced increased T regs. Furthermore, the upregulation of CD141 and GARP as well as generation of T regs were both compromised in RA exposed DCs from aged subjects. Recent reports from the literature also suggested that CD141 may be anti-inflammatory [30, 31]. In addition, GARP is already associated with tolerance on T cells. Therefore, we explored whether there was a correlation between the two phenomena by determining the cytokine secretion and T reg induction capacity of CD141 and GARP^+^ DCs. Next, we purified CD141 high, GARP^+^ (hereafter referred to as CD141^hi^) and CD141low, GARP^−^ (hereafter referred to as CD141^lo^) DC populations. Purified CD141^hi^ and CD141^lo^ DCs were subsequently cultured with CD4^+^T cells and secretion of IFN-γ and IL-10 and generation T regs was determined as described. Figure 4A depicts the flow chart for the experimental set up in this figure. Culture of both CD141^hi^ and CD141^lo^ DCs with T cells resulted in comparable levels of IFN-γ secretion (Figure 4B), however, the secretion of IL-10 from T cells was significantly (p<0.05) increased in CD141^hi^ DC group compared to CD141^lo^ DCs group (Figure 4C). CD141^hi^ DCs also induced significantly (p<0.05) higher percentages of T regs compared to CD141^lo^ DCs (Figure 4D & E).

To further confirm that indeed, the CD141^hi^ DCs are anti-inflammatory and tolerogenic, CD141^hi^ and CD141^lo^ DCs were activated with TLR-2 ligand, Pam-3-Cys (Pam) and the upregulation of activation markers and secretion of cytokines was determined. The activation of CD141^lo^ DCs with Pam led to significant (p<0.05) upregulation of costimulatory molecules, CD80 and CD86 on DCs compared to unstimulated controls (Figures 3F & G). In contrast, there was no upregulation of CD80 and a downregulation of CD86 on Pam-activated CD141^hi^ DCs (Figures 4F & G). The upregulation of antigen presenting molecule, HLADR was also downregulated on Pam-activated CD141^hi^DCs while it displayed significantly (p<0.05) enhanced expression on Pam-activated CD141^lo^ DCs compared to their respective controls (Figure 4H). Assay of cytokines also revealed a similar picture since the Pam-stimulated CD141^hi^ DCs displayed reduced production of pro-inflammatory cytokine, TNF-α compared to Pam-stimulated CD141^lo^ DCs (Figure 4I). More importantly, CD141^hi^ DCs secreted significantly (p<0.05) increased levels of anti-inflammatory cytokine, IL-10 both at the basal level and after activation with Pam compared to their CD141^lo^ counterparts (Figure 4J).

In summary, CD141^hi^ DCs are tolerogenic/anti-inflammatory compared to CD141^lo^ DCs. Since CD141^hi^ DCs also express GARP therefore reduced CD141 and GARP expression by RA exposed DCs from aged subjects may be responsible for impaired induction of Tregs.

### CD141^hi^ DCs from aged subjects are impaired in their capacity to induce T regs

**Figure 5:**
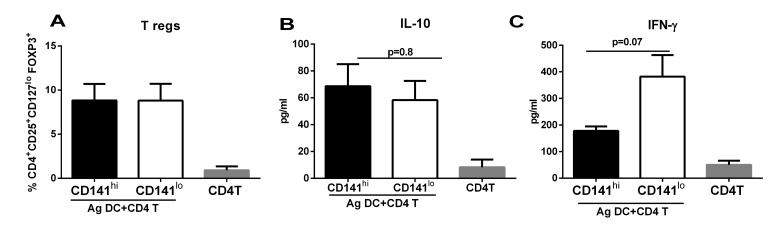
CD141^hi^ DCs from aged subjects are impaired in their capacity to induce T regs DCs from aged subjects were stimulated with RA and sorted into CD141^hi^, GARP^+^ (CD141^hi^) and CD141^lo^, GARP^−^ (CD141^lo^) populations. The 2 sorted DC subsets were then cultured with allogenic CD4 T cells. Bar graphs depict- (**A**) the percentage of CD4^+^CD25^+^CD127^lo^ FoxP3^+^ T reg cells; (**B**) the level of IL-10 secretion; (**C**) IFN-γ secretion. Figures are mean ^+^/− S.E. of 6 different experiments.

Though the percentages of GARP^+^, CD141^+^ DCs was reduced in RA treated DCs from aged subjects it remained to be determined whether these cells were functional. Therefore, we examined the T reg inducing capacity of CD141^hi^ DCs from aged subjects. As is evident from figure 5A, CD141^hi^ DCs from aged subjects did not induce significantly (p>0.05) increased levels of Tregs compared to CD141^lo^ DCs which was the case in DCs from young subjects (Figure 4D). There was no increase in the induction of IL-10 (Figure 5B), however, the secretion of IFN-γ was reduced though the decrease was not significant (p=0.07) (Figure 5C). This was in contrast to young DC where we had observed significantly increased (p<0.05) secretion of IL-10 (Figure 4C) and reduced secretion of IFN-γ (Figure 4B) from T cells cultured with CD141^hi^ DCs.

Together, these data suggest that though the CD141^hi^ DCs from aged subjects display impaired capacity to induce Tregs and IL-10 compared to young; nevertheless, they do possess some degree of anti-inflammatory capacity since the secretion of IFN-γ is reduced from T cells cultured with CD141^hi^ DCs from aged individuals.

### Circulating myeloid DCs (mDCs) display similar response to RA as monocyte derived DCs

Next, we determined whether circulating myeloid DC (mDC) subsets also upregulated CD141 and GARP expression on exposure to RA. Circulating mDCs are subdivided into two phenotypically distinct subsets of CD11c^+^ DC, defined by their expression of CD1c (BDCA-1), and CD141 (BDCA-3, thrombomodulin) [32]. CD1c^+^ and CD141^+^ DCs each have unique gene expression profiles and differ in their expression of pattern recognition receptors (PRRs), cytokine production and T cell polarizing abilities [33, 34].

CD141^+^ DCs possess a superior capacity to cross-present antigen to CD8^+^ cytotoxic T lymphocytes which is required for immunity against tumors and viruses [33]. CD1c^+^ DCs on the other hand are considered more efficient in priming CD4 T cell responses [35]. Interestingly, the numbers of CD141^+^ DCs is reported to be higher in mucosal tissues.

**Figure 6:**
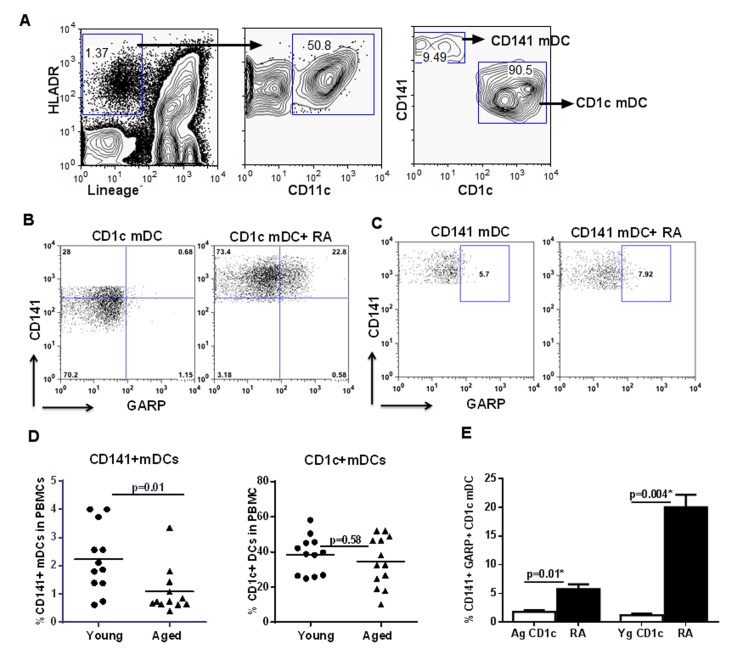
Circulating myeloid DCs (mDCs) display similar response to RA as monocyte derived DCs Purified mDCs from aged and young were exposed to RA for 24h. (**A**) Zebra plots depict the subsets of CD141^+^ and CD1c^+^ mDC populations in the young PBMCs as determined by flow cytometry. Dot plots depict the upregulation of CD141 and GARP on- (**B**) CD1c^+^ mDC subset; (**C**) CD141^+^ mDC subset in the young subjects. Figures are representative of 6 different experiments. (**D**) Dot blot depicts the percentage of CD141^+^ and CD1c^+^ mDC subsets in the circulation of aged and young subjects. n=12. (**E**) Bar graph depicts the percentage of CD141^+^, GARP^+^ CD1c mDC from aged and young subjects after treatment with RA. Figures are mean ^+^/− S.E. of 6 experiments with different aged and young subjects.

Negatively purified mDCs from young were exposed to RA. RA exposure also led to the upregulation of both CD141 and GARP on circulating mDCs. Remarkably, the upregulation of CD141 and GARP was more prominent on the CD1c^+^mDC subset (Figure 6B). Exposure of CD141^+^mDC to RA resulted in slightly increased GARP expression but did not lead to further upregulation of CD141 expression (Figure 6C).

Next, we compared the response of mDCs from aged and young individuals to RA. Interestingly, reduced expression of CD141 on DCs in aged seemed to be universal since when we compared the percentages of CD1c^+^ and CD141^+^ mDC subsets in the circulation of aged and young subjects, there was a significant (p<0.05) reduction in the percentage of CD141^+^mDC subset in the elderly compared to the young subjects (Figure 6D). The percentage of CD1c^+^ mDC subset on the other hand was comparable between the two age groups. Further experiments therefore focused only on CD1c^+^ subset. Similar to monocyte derived DCs, CD1c^+^ mDC from aged individuals also displayed reduced expression of CD141 and GARP compared to DCs from the young, on exposure to RA (Figure 6E).

Altogether, these data suggest that DCs from aged, circulatory and monocyte-derived display reduced response to RA.

## DISCUSSION

The current study reports a previously uncharacterized mechanism of Treg induction by RA exposed DCs which is defective in aged subjects. RA exposure leads to the upregulation of CD141 and GARP on DCs and that the CD141^+^ and GARP^+^ subset of RA stimulated DCs is the one which induces T regs. Furthermore, we also demonstrate that the response of DCs to RA is significantly compromised in the elderly resulting in reduced upregulation of CD141 and GARP and subsequent impaired ability to induce T regulatory cells. Previous studies have demonstrated that RA treatment of murine DCs induces the expression of CD103 on DCs which generates Tregs [13]. Only a few studies have investigated the effect of RA on CD103 expression on human DCs. One such study shows upregulation of CD103 on DCs when they are differentiated from monocytes in the presence of RA[10]. In contrast, another study by Chatterjee et al. [36], reported a very low level upregulation of CD103 on DCs after RA exposure. This discrepancy could be due to the difference in experimental conditions since the first study differentiated the DCs from monocytes in the presence of RA while the second study exposed the DCs to RA after differentiation. Our results are similar Chatterjee et al. since we also did not observe significantly upregulation of CD103 on DCs after exposure to RA (Figure 2B & C).

Haniffa et al [25] have reported that human CD141 DCs are akin to murine CD103 DCs. We did find that CD141 expression was upregulated on DCs after RA exposure both at the level of mRNA and protein (Figure 2A & B). This expression was significantly reduced in RA treated DCs from aged subjects. CD141/BDCA-3 is a marker used to define a subset of DCs but its function on DCs is not known. Studies indicate that CD141 may function as an anti-inflammatory molecule [30, 31]. Transgenic mice with deleted transmembrane domain of CD141 displayed stronger inflammatory reaction after lipopolysaccharide (LPS) stimulation. [37] These mice also displayed leukocytes accumulation in the lungs after inhalation of gram-negative bacteria and increased mortality in endotoxin-induced sepsis. In another study with murine bone-marrow derived dendritic cells, the authors observed increased phosphorylation of several cell cycle kinases in thrombomodulin (+) DCs compared to thrombomodulin (−) DCs, while phosphorylation of kinases involved with pro-inflammatory cytokine signaling was reduced [31]. Production of IL-10 was also increased in thrombomodulin (+) dendritic cells.

In addition to CD141, we also observed enhanced expression of GARP on DCs after exposure to RA (Figure 2A & B). GARP is transmembrane receptor which tethers and stabilizes the expression of LAP/TGF-β1 on the surface of cells [27, 28]. Surface bound LAP/TGF-β1 has been demonstrated to be important for generation of Tregs. Several cell types express LAP including DCs, however, GARP expression has been studied primarily on activated Tregs and platelets where it is involved in T cell differentiation and tolerance induction [27, 28]. Two recent studies have reported GARP expression of MSCs [29] and hepatic stellate cells [38] where also it has been demonstrated to inhibit T cell function. Knocking down GARP on MSCs reduced their immuno-suppressive capacity. Soluble GARP has also been reported to be potent suppressor of T cell proliferation [39]. Altogether, our data suggests a novel mechanism of mucosal tolerance whereby RA secreted by epithelial DCs or DCs in the mucosa induces the expression of multiple immune suppressive molecules such as CD141, GARP, on DCs which render the DCs tolerogenic. Our results support this notion as we found increased secretion of IL-10 by CD141^hi^ (CD141^hi^ and GARP^+^) DCs compared to CD141^lo^ DCs. In addition, the CD141^hi^ DCs induce significantly higher percentages of Tregs compared to CD141^lo^ DCs reiterating the anti-inflammatory nature of CD141^hi^ DCs. The correlation between CD141, GARP expression and Treg has not been explored until now. Our results suggest that increased Treg generation is probably a consequence of increased IL-10 secretion by CD141^hi^ DCs (Figure 4).

Further confirmation regarding the role of CD141 and GARP in induction of Tregs is obtained by our results with DCs from aged subjects who display significantly reduced expression of CD141 and GARP and are deficient in their capacity to induce T regs (Figure 1 & 2). Interestingly, the reduced percentages of CD141^hi^, GARP^+^ DCs from aged were also deficient in inducing IL-10 and T regs but did display some capacity to reduce IFN-γ(Figure 5). The intrinsic defect in DCs from aged to produce and induce IL-10 is something we have observed in our earlier studies [21]. However, in lieu of IL-10 some other as yet unexplored anti-inflammatory mechanisms seem to operate in DCs from aged subjects.

Interestingly, the defect in CD141 expression in DCs from aged individuals does not seem limited to stimulation of DCs as we also found decreased percentages of CD141^+^ mDC subset in the circulation of aged subjects (Figure 6D). Human CD141^+^ DCs are homologous to mouse CD8α^+^ DCs which are important for anti-viral and anti-tumor immunity [31, 33]. Reduced CD141^+^ mDC subset may be responsible for the increase in viral infections and tumors in the elderly. Furthermore, CD141^+^ DCs are considered the human counterpart of CD103^+^ murine DC subset found in the mucosa [25]. Our observation that CD1c^+^ mDCs can upregulate CD141^+^ on RA exposure suggests that the enhanced numbers of CD141^+^ DCs observed in the human mucosa may simply be CD1c^+^ mDCs that have upregulated the expression of CD141 because of abundance of RA in the mucosa.

**Figure 7:**
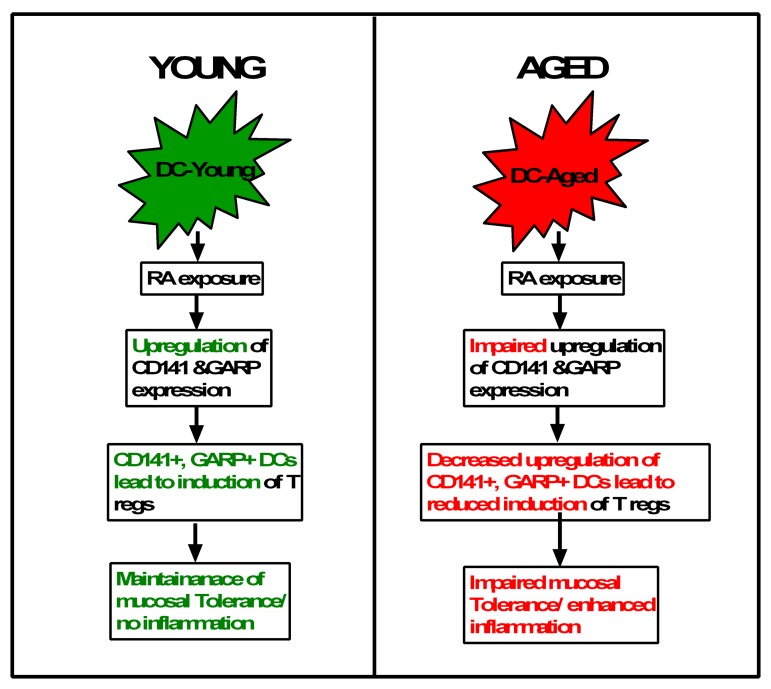
Flow chart representation of the main conclusions of the paper RA present in the mucosa in known to induce tolerance via induction of T regs. However, the mechanisms of T reg induction are not understood. Here we outline a novel mechanism of induction of T regs by RA which is impaired in the elderly. Exposure of young DCs to RA induces the upregulation of expression of CD141 and GARP/LRRC32 on DCs. The culture of CD141hi GARP+ DCs with CD4 T cells leads to the induction of T regulatory cells and IL-10 secretion versus the CD141lo GARP− DCs. This suggests that RA induces T regs via CD141 and GARP expression which maintains mucosal tolerance and prevents inflammation. In contrast to young, DCs from aged subjects are impaired in their capacity to upregulate CD141 and GARP after treatment with RA. This reduces their capacity to induce T regs and maintain mucosal tolerance and control inflammation.

In conclusion (Figure 7), compared to young, DCs from aged display a deficiency in their response RA. There is impaired upregulation of CD141 and GARP on RA-stimulated DCs from aged individuals. The diminished expression of CD141 and GARP results in a decreased capacity of aged DC to induce Tregs. Our results there-fore suggest a novel mechanism that can cause severe and chronic inflammation in mucosa of the elderly, thus enhancing their susceptibility to mucosal infections and diseases. To our knowledge this the first report of RA inducing the expression of CD141, GARP on DCs and the effect of age on the response of DCs to RA.

## MATERIALS AND METHODS

### Blood donors

Peripheral blood samples were obtained from healthy aged and young volunteers. The young donors were between 20 and 35 years of age and aged donors between 60 and 90 years. Elderly subjects belong to middle-class socioeconomic status and are living independently. Description of the cohort is provided in Table I. This study was approved by the Institutional Review Board of the University of California (Irvine, CA).

**Table 1:** Description of the aged and young cohorts

Young n=30	Aged n=30	
Age (range)	25(20-32)	75(60-90)
Female	19(63%)	23(76%)
**Comorbidities**		
Osteoarthritis	0	18(60%)
Hypertension	0	16(53%)
Dyslipidemia	0	5 (16%)
Diabetes	0	0
**Medications**		
Vitamins	0	24 (80%)
Antioxidants	0	20 (66%)

### Stimulation of DCs with RA

Monocyte derived DCs from aged and young subjects were prepared essentially as described[21]. Collected DCs were cultured in serum free, AIM V medium with all trans Retinoic acid (RA, Sigma-Aldrich) at a concentration of 1 μM/ml. 24-48h later, the DCs were collected and stained for surface markers CD11c, CD141, CD103, LAP, GARP using specific antibodies and respective isotypes (CD141, CD103 from BD Bioscience, San Jose, CA; CD11c and GARP from Miltenyi Biotech and LAP from RnD systems). RA stimulated DCs were also used to culture with T cells.

### DC-T cell co-culture

DCs exposed to RA were cultured with purified, allogenic CD4 T cells at a ratio of 1:10 for 5 days. Subsequently, the supernatants were collected and assayed for cytokines, IL-10 and IFN-γ. Cells collected were stained for CD4^+^CD25^+^CD127^lo^

FoxP3^+^ T regulatory cells (Tregs) using specific antibodies.

### Staining for mDC subsets in circulation

PBMCs from aged and young subjects were stained with blood DC enumeration kit (Miltenyi Biotech) to determine the percentages of CD141^+^ and CD1c^+^ myeloid DC subsets in circulation.

### CD141 and GARP co-localization

DCs were treated overnight with RA and subsequently stained with CD11c, CD141 and GARP. All samples were acquired on an ImageStream^X^ imaging cytometer, X60 magnification; with low flow rate/high sensitivity using INSPIRE software. A minimum of 10,000 events were collected. Cells were selected for positivity in, Ch 2 (CD11c FITC) and expressing Ch3 (CD141 PE) and Ch11 (GARP APC).

### CD141^hi^ and CD141^lo^ DCs

DCs from aged and young subjects were exposed overnight to RA (1μM/ml). CD141^+^ DCs were purified either by positive selection using magnetic beads (Stemcell, Vancouver, Canada) or by flow sorting by FACSAria. The purified CD141^hi^ and CD141^lo^ fractions were cultured with purified CD4 T cells as described above. Supernatants collected were assayed for IFN-γ and IL-10 by ELISA. In a separate set of experiments, the purified CD141^hi^ and CD141^lo^ MoDC fractions from young subjects were stimulated with TLR-2 ligand, PAM-3Cys for 24h in AIMV. Subsequently, the cells were collected and stained for DC activation markers, CD80, CD86 and HLADR. Supernatants collected were assayed for TNF-α and IL-10 by ELISA.

### Gene expression

DCs from aged and young were exposed to RA for 2h. RNA was extracted using Tri reagent. Real time PCR was performed using validated specific primers for CD141 (*THBD*), CD103 (*ITGAE*), GARP(*LRRC32*) and LAP (Real time primers LLC, Elkins Park, PA). GAPDH was used as the housekeeping gene.

### Statistical analyses

Statistical analysis was performed using Graph pad prism (GraphPad Inc., San Diego, CA). Differences between unstimulated and stimulated conditions were tested using paired t test. Differences between aged and young subjects DC functions were tested using Mann-Whitney test. A *P*-value of <0.05 was considered statistically significant.

## SUPPLEMENTARY FIGURE


