# Supplementary material for: Retinoic acid treated human dendritic cells induce T regulatory cells via the expression of CD141 and GARP which is impaired with age
Source: Aging (Albany NY). 2016 May 30;8(6):1223–35. doi: 10.18632/aging.100973 (PMC4931828; doi:10.18632/aging.100973)
Supplement: Supplementary file 1 [file aging-08-1223-s001.pdf]

37. Conway EM, Van de Wouwer M, Pollefeyt S, Jurk K, Van Aken H, De Vriese A, Weitz JI, Weiler H, Hellings PW, Schaeffer P, Herbert JM, Collen D and Theilmeier G. The lectin-like domain of thrombomodulin confers protection from neutrophil-mediated tissue damage by suppressing adhesion molecule expression via nuclear factor kappaB and mitogen-activated protein kinase pathways. The Journal of experimental medicine. 2002; 196:565-77.
38. Li Y, Kim BG, Qian S, Letterio JJ, Fung JJ, Lu L and Lin F. Hepatic Stellate Cells Inhibit T Cells through Active TGF-beta1 from a Cell Surface-Bound Latent TGF-beta1/GARP Complex. Journal of immunology. 2015; 195:2648-56.
39. Hahn SA, Stahl HF, Becker C, Correll A, Schneider FJ, Tuettenberg A and Jonuleit H. Soluble GARP has potent antiinflammatory and immunomodulatory impact on human CD4(+) T cells. Blood. 2013; 122:1182-91.

SUPPLEMENTARY FIGURE

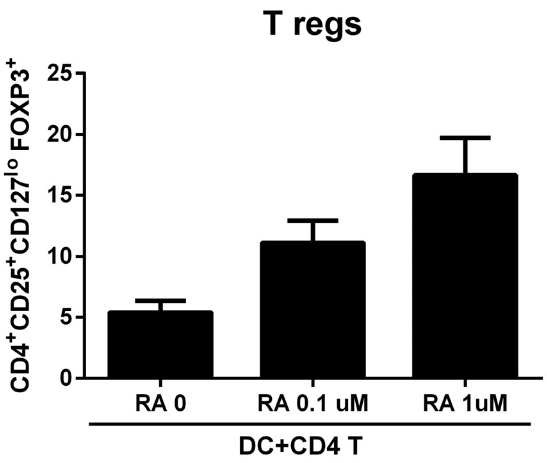

Supplementary Figure 1. Determining of optimal concentration of RA. DCs were exposed to RA at concentrations ranging from 0-10μM/ml for 24h. Subsequently the DCs were collected and cultured with purified, allogenic CD4+ T cells for 5 days. The T cells were collected at the end of the culture and stained for CD4, CD25, CD127 and Foxp3. Bar graph depicts the % of Tregs in the culture as determined by flow cytometry. RA concentation of 1 μM was found to be optimal. RA concentration of 10 μM was found to be toxic to DCs. Data is mean +/- S.E. of 4 experiments.
